# Preformulation Studies of Zidovudine Derivatives: Acid Dissociation Constants, Differential Scanning Calorimetry, Thermogravimetry, X-Ray Powder Diffractometry and Aqueous Stability Studies

**DOI:** 10.3797/scipharm.1105-04

**Published:** 2011-07-25

**Authors:** Mónica A. Raviolo, Margarita C. Briñón

**Affiliations:** Departamento de Farmacia, Facultad de Ciencias Químicas, Ciudad Universitaria, Universidad Nacional de Córdoba, 5000 Córdoba, Argentina

**Keywords:** AZT derivatives, Acid dissociation constants, DSC and TG, X-Ray Powder Diffractometry, Aqueous stability

## Abstract

As part as of the preformulation studies of new 5′-OH derivatives of zidovudine, compounds **2–6**, their acid dissociation constants, Differential Scanning Calorimetry (DSC) and Thermogravimetry (TG) curves, X-Ray Powder diffractograms and aqueous stability are reported. A sensitive technique such as differential scanning potentiometry was used to determine the pKa constants of the above mentioned compounds. In addition, pKa values were calculated from theoretical methods, and no significant differences with those of experimental ones were observed. X-Ray Powder Diffractometry data demonstrated that compounds **2–4** were crystalline while **5** and **6** were amorphous. DSC analysis indicated that all of them presented an exothermic decomposition peak above 150 °C which is accompanied by a weight loss in the respective TG curves. The stability of these compounds in aqueous medium at different pH values was investigated, using a validated High Performance Liquid Chromatography (HPLC) method, which demonstrated to be rapid, selective, sensitive, accurate and stability-indicating. Good recovery, linearity and precision were also achieved. For all compounds the aqueous hydrolysis followed a pseudo-first-order kinetics, depending on pH and the union existing between AZT and the associate moiety. The hydrolysis was catalyzed by hydroxide ion in the 7.4–13.2 pH range, while all compounds exhibited pH-independent stability from acidic to neutral media (pHs 1.0–7.4).

## Introduction

Acquired immunodeficiency syndrome (AIDS) results from infection by the retrovirus, human immunodeficiency virus (HIV-1) [1]. 3′-Azido-3′-deoxythymidine (zidovudine, AZT, **1**), the first clinically successful nucleoside analog for the treatment of AIDS, is a potent nucleoside reverse transcriptase inhibitor (NRTI) of HIV-1 replication [2], particularly when used in combination with other drugs, such as other NRTIs, non-nucleoside reverse transcriptase and protease inhibitors [3]. Although nucleoside analogues continue to play a prominent role as antiviral agents [4], their therapeutic potential is often hampered by their poor biopharmaceutical properties [5]. Therefore, as part of our ongoing efforts to search for novel antiviral agents, we developed AZT derivatives with anti HIV activity, modifying the 5′-OH position of AZT which could form a reservoir of the drug in different human tissues, thus requiring less frequent dosing [6–11].

Parameters obtained from preformulation studies are essential to understand the biological activity of drugs, by depicting a more accurate picture of the macromolecule-ligand binding interaction. Through these studies, it is possible to derive valuable information on different properties of drugs [12–14]. Thus, the acid dissociation constant (pKa) is a useful physicochemical parameter to evaluate the extent of ionization of functional groups related to pH, often of vital significance to understand the pharmacokinetic and pharmacodynamic behavior of a drug substance [15]. Possible lack of sharpness of the inflection points of the pH-metric titration to establish the pKa of ionizable groups in aqueous solution is the main limiting factor in conventional aqueous acid-base potentiometry, which depends on the strength and concentration of the sample species as well as on the presence of interfering substances. Differential Scanning Potentiometry (DSP) yields areas as analytical responses, thereby enhancing its sensitivity for determining weak acids and bases in aqueous systems [16, 17].

Differential Scanning Calorimetry (DSC), Thermogravimetry (TG) and X-ray Powder Diffractometry (XRPD) are suitable and widely used techniques for the characterization of the solid state of a substance for preformulation studies, while stability studies are also relevant to evaluate promptly any instability during drug discovery and development, to determine whether the whole molecule or their degradation products, as occurs with prodrug compounds leading to parent drugs, are responsible for the observed activity [18].

**Fig. 1.**
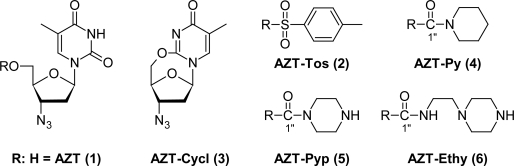
Chemical structures of AZT (**1**) and its derivatives (2–6).

Thus, the aim of this work was to describe relevant preformulation determinations for **2**–**6** [6] (Fig 1), such as their acid dissociation constants (pKa), DSC and TG curves, XRPD profiles and aqueous stability. These studies were performed since they are important early requirements for novel compounds in drug research. Previously, we also have reported the lipophilicity and liposome permeability of **2–6** [7, 19].

## Results

### pKa Determination

In the differential scanning potentiometry (DSP) methodology, acid and basic groups generate negative and positive areas, respectively [16, 17]. Thus, the NH acidic groups of AZT and thymidine were used as reference to validate the DSP, since their pKa values had been previously reported [20]. The linear relationship between the amount of titrated sample and the resulting area was first investigated for an interval of 0.04–0.16 mmol L^−1^; 0.12 mmol L^−1^ was selected as an appropriate concentration for pKa assays by DSP (eq. 1), where C represents the mmol L^−1^ concentration.

Eq. 1. $$\begin{array}{l} \text{Area }=\;21.29\;(\pm \;3.32)\text{ C }+\;0\text{.26 (}\pm \;0.37)\\ \text{r }=\;0\text{.994};\text{ SD }=\;0\text{.115};\text{ n }=\;6 \end{array}$$

Then, using compounds with different pKa values (sulphanilamide = 2.36 and 3.57; procaine hydrochloride = 5.20; lidocaine = 7.86; erytromycine = 8.80; *p*-aminobenzoic acid = 9.10 and potassium hydrogen phthalate = 11.05), the regression analysis of their areas vs their pKa values was carried out (eq. 2).

Eq. 2. $$\begin{array}{l} \text{Area }=\;1.08\;(\pm \;0.09)\;\text{pKa}\;-\;2\text{.13}\;(\pm \;0.67)\\ \text{r }=\;0\text{.998};\text{ SD }=\;0\text{.271};\text{ n }=\;7 \end{array}$$

**Fig. 2.**
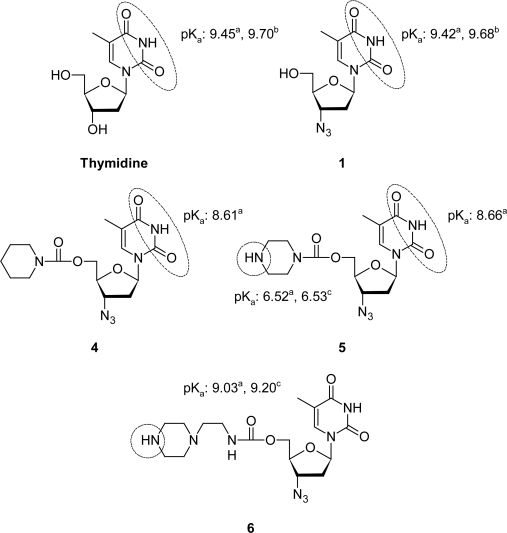
Thymidine, **1** and **4–6** derivatives with their ionizable groups and pKa values. ^a^ Experimental values; ^b^ previously reported experimental values; ^c^ calculated values employing Taft equations.

Once the experimental areas for thymidine, **1** and **4**–**6** were obtained, their pKa values were calculated using eq. 2 as shown in Figure 2.

To confirm the values obtained for the aliphatic amines of compounds **5** and **6**, predictive methods were followed by applying the Taft equation for protonated secondary amines (R_1_R_2_NH_2_^+^, pKa = 10.59 – 3.23 Σσ*, where σ* is the electronic Taft constant [21]) as previously reported for a related compound [22]. Figure 2 also shows the pKa values calculated for **5** and **6**.

### DSC and TG data

**Fig. 3.**
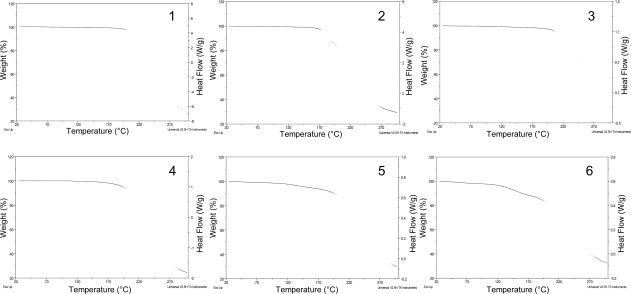
DSC (- - - . ) and TG (—) curves of AZT (**1**) and their derivatives (**2–6**).

The DSC curves of **2** and **4** (Figure 3) exhibited, in the temperature range of 25–150 °C, endothermic peaks at 115.8 and 112.8 °C, respectively (extrapolated onset temperature, T onset) due to fusion, while those of compounds **5** and **6** showed a small endothermic peak corresponding to a glass transition at 35.1 and 42.5 °C, respectively (T onset). Compound **3** only displayed a DSC exothermic decomposition peak at 215 °C. Compounds **2, 4, 5** and **6** also presented an exothermic decomposition peak above 150 °C, which is accompanied by a weight loss in the respective TG curves (Figure 3).

### X-Ray powder diffractometry (XRPD)

**Fig. 4.**
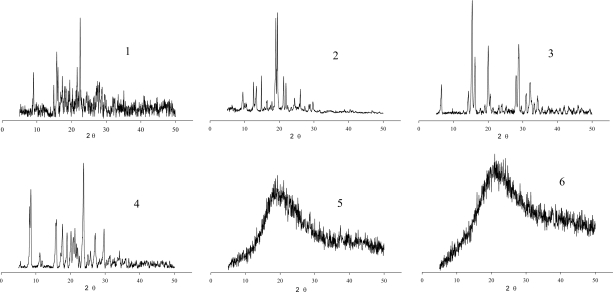
X-Ray diffraction patterns of AZT (**1**) and their derivatives (**2–6**).

XRPD analysis provides an accurate identification of crystalline and amorphous solid phases. The XRPD patterns of AZT and their derivatives **2**–**6** are depicted in Figure 4. The diffractograms of **1**–**4** showed various peaks, indicating that they are solid compounds; whereas in **5** and **6**, the fine and sharp peak profiles disappeared, yielding a broad band, typical of an X-ray amorphous solid.

### Aqueous stability

Stability in buffers of pH values corresponding to gastrointestinal tract (GI) and plasma media is important for drug candidates. The pH of GI varies from acidic (pH 1.2) in the stomach to basic pH (pH 8.0) in the small and large intestine. Therefore, acceptable stability in the pH range along the aqueous GI and plasma media is essential to achieve good oral bioavailability. Before determining the kinetics of the compounds assayed, it is necessary to validate the analytical method.

### Validation method

The standard graphs for all compounds were satisfactorily described by least-square linear regression. Calibration graphs were constructed using the areas of the chromatographic peaks (triplicate injections) obtained at eight different concentrations, equally distributed in the range from 1 × 10^−6^ to 1 × 10^−4^ mmol mL^−1^ for all compounds. The limits of detection (LOD) and quantification (LOQ) for the compounds were calculated with the *3s criterion* and the *10s criterion* (three and ten times the standard deviation of the lowest concentration solution included in the calibration divided by the slope of the calibration curve) using a series of 10 solutions containing a low concentration of each compound.

Precision, defined as the relative standard deviation (RSD %), was determined by intra- and inter-day assays at a medium concentration, according to the calibration graph ranges (Table 1). The intra-day precision was calculated by measuring the areas of the peaks obtained from 10 injections of a test solution on the same day. The inter-day analysis involved the average of ten measurements of intra-day values calculated during ten days over a 3-month period at the same concentrations by different analysts and in different equipment.

Table 1 shows the slopes, intercepts, regression coefficients (*r*) of the calibration graphs, LOD and LOQ, as well as the intra- and inter-day precision (RSD %) parameters. As described in this table, satisfactory regression coefficients (r ≈ 0.999) for the calibration graphs and the percentage of the RSD were obtained.

### Identification of Degradation Products

**Fig. 5.**
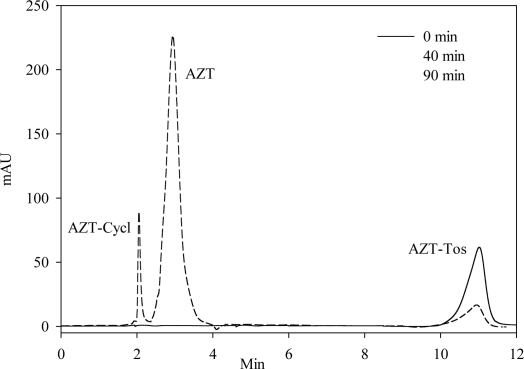
Chromatograms corresponding to AZT-Tos and their degradation products (AZT and AZT-Cycl), using system A as eluting mobile phase. The chromatograms correspond to samples at different times, 0, 40 and 90 min at pH 9.4.

The stress kinetic for degradation of **2–6** was evaluated at 50, 70 and 90 °C in aqueous media at different pH buffers (1.0, 2.6, 7.4, 9.4 and 13.2). In all cases, identification of each intact compound and AZT (the main degradation product) was performed using HPLC, by comparing their HPLC retention times (t_R_) with those of authentic samples. Three solvent systems, A, B and C, were used to obtain an optimal separation of the compound peaks. For solvent system A, t_R_ peaks appear at 3.0 min for **1**, 11.0 min for **2,** 2.0 min for **3** and 10.8 min for **4**; for solvent system B, t_R_ peaks appear at 4 min for **1**, 4.8 min for **5** and 5.2 min for **6**; and for solvent system C, t_R_ peaks appear at 5.0 min for **1** and 3.6 min for **3**. Hence, good peak separations were obtained enabling identification of compounds **2–6**. As an example, Figure 5 shows the HPLC chromatograms of AZT-Tos (**2**) and their degradation compounds (AZT, **1** and AZT-Cycl, **3**), using solvent system A. The chromatograms correspond to samples of different times: 0, 40 and 90 min of the test at pH 9.4.

### Stability Studies

The aqueous stability studies for **2–6** were carried out at different pH media (1.0, 7.4, 9.4 and 13.2) to determine whether the whole molecule or its degradation products were responsible for the anti-HIV activity observed [6]. These studies were carried out at 60 °C, and showed a linear relationship between the logarithmic concentration of the intact drug and the storage time, thereby indicating a pseudo-first-order degradation kinetics at the pHs analyzed. Table 2 lists the pH media, experimental rate constants (k_obs,_ when it could be determined) and their corresponding half-life times (t_½_).

As expected, analysis of Table 2 shows that AZT proved to be stable at the studied pH media. All AZT derivatives, except **2**, led to AZT as the degradation product, while **2** at pHs 7.4 and 9.4 yielded **3** as the degradation product. This is not surprising since **3** was obtained from **2** in an alkaline environment [6] resulting in greater instability for increasing pH. On the other hand, **3** proved to be more stable than **2** at pH 7.4.

The 5′-*O*-carbamate compounds **4**–**6** showed degradation reactions at an alkaline pH of 13.2, with k_obs_ values of 1.04 × 10^−3^, 3.23 × 10^−3^ and 1.96 × 10^−3^ min^−1^, respectively due to a base catalysis with a decreasing stability of **4** > **6** > **5**. Similar results were observed by Norberto *et al.* for related compounds at different pH values [23].

## Discussion

The validation of DSP technique for determining the pKa of **2–6** was carried out using two reference compounds (AZT and thymidine). Then the pKa of the NH group of the pyrimidinic base of **4** and **5** was calculated. Similar values to that of AZT were obtained, which indicated that DSP is adequate for determining pKa values for this type of compounds.

The acid dissociation constants for aliphatic amines of **5** and **6** were also determined, with a pKa 9.03 for the amine moiety of **6** showing a higher basicity than that of the corresponding amine group of **5** (pKa 6.52). This occurred since this NH group in **6** was far from the carbamate group and expected to influence on its ionization. The pKa values calculated from Taft method [21] or the amine groups of **5** (pKa 6.53) and **6** (pKa 9.20) showed a good correlation with those of the experimental ones (Figure 2).

Under the crystallization conditions used, compounds **2** (from EtOH), **3** [from EtOAc/(CH_3_)_2_CO] and **4** [from (CH_3_)_2_CO/MeOH] crystallized while **5** and **6** [from (CH_3_)_2_CO/MeOH] did not. On the other hand, DSC and TG data demonstrated that compounds **2** (115.8 °C) and **4** (112.8 °C) showed sharp melting peaks, typical of crystalline solids, and compounds **5** and **6** exhibited glass transitions at 35.1 and 42.5 °C, respectively, discarding the possibility that they crystallized as microcrystalline solids.

Regarding the crystallization properties of the studied compounds, it is interesting to note that a similar behavior was observed for other AZT esters [11,24], i.e. under similar crystallization conditions to those used with **2–6,** some crystallized and others not.

In aqueous stability studies, all derivatives decomposed to give AZT, the parent drug, following pseudo first-order kinetics. It should be noted that compound **2** at neutral or alkaline pH was first degraded to **3**, before giving AZT in a second step. Derivatives **2** and **3** demonstrated different stabilities depending on the pH environment, while 5′-*O*-carbamates **4–6** only decomposed in a strong alkaline media (pH 13.2). Previously, we reported that compound **2** and carbamates **4**–**6** were stable in rat plasma since they were able to display conformations that did not allow their access to the catalytic cavity of the enzyme able to hydrolyze them [6]. The carbamate derivatives have demonstrated a strong bond between AZT and the associated moieties, preventing hydrolysis and parent compound regeneration. For this reason, compounds **2**, **4**–**6** could not act as prodrugs, as they lacked the necessary activity found in previous *in vitro* studies [6]. The aqueous stability of the cyclic derivative **3** was different from that of the carbamates. Thus, it could be degraded in highly acidic (pH 1, t_1/2_ = 128 min) or alkaline (pH 9.4, t_1/2_ = 124.2 min) media, thereby obtaining high stability in the neutral medium (pH 7.4, t_1/2_ = 4750 min). Moreover, this compound showed stability in rat plasma for 30 h of incubation at 37 °C, which implied that it was also acting as a drug, since this derivative was found to have anti-HIV activity and no cytotoxicity [6].

## Experimental

### Materials

Methanol (MeOH) HPLC grade, was purchased from Baker Co. Dimethylsulfoxide (DMSO) and tetrahydrofurane (THF), both of analytical grade, were provided by Cicarelli Co (Buenos Aires, Argentina). The water used in HPLC analyses and in all studies was of Milli-Q grade, and mobile phases were filtered through Millipore^®^ filters Type FH (4.5 μm) and degassed under vacuum. All other chemicals and solvents were of analytical grade. AZT was generously supplied by Filaxis (Buenos Aires, Argentina). Compounds **2–6** were prepared as reported [6].

### pKa - Differential Scanning Potentiometry (DSP)

A conventional pH-meter (GLP 21, Crison Instrument) with an Ag/AgCl-reference electrode was used. NaOH was added at 0.1 mL increments, with its corresponding pH value recorded from the average of two sample determinations using DMSO as the cosolvent (2.5 %) at 25 °C. The areas were calculated using the Origin 7 program. For the reference graph, an aliquot of 10 mL of HCl (0.1 mol L^−1^) and 0.5 mL of DMSO was titrated with NaOH (0.1 mol L^−1^); for the sample graphs, aliquots (10 mL) of HCl (0.1 mol L^−1^), 0.5 mL DMSO and 0.12 mmol for each compound were titrated with NaOH (0.1 mol L^−1^). The volume of each solution was completed to 20 mL with Milli-Q water before titration.

### Differential Scanning Calorimetry (DSC), Thermogravimetric Analysis (TG) and X-Ray Powder Diffractometry (XRPD)

DSC measurements were carried out using a 2920 modulated DSC (TA Instrument). The temperature axis and the cell constant of the DSC were calibrated with indium. Weighed samples (1.5–3 mg, C-33 Microbalance, Cahan) were scanned in covered aluminium (Al) pans under a dynamic dry nitrogen atmosphere (50 mL min^−1^) starting at 25 °C and raising the temperature in 5 °C steps until reaching 300 °C. TG measurements were performed with a TA Instrument Hi-res TG 2950 (TA Instrument). Samples (1.5–3 mg) were weighed in Al pans, starting at 25 °C and raising the temperature in 5 °C steps until reaching 300 °C, under a dynamic dry nitrogen atmosphere (50 mL min^−1^). The Universal Analysis Version 2.5H software was used for both measurements. The XRPD patterns were determined at around 20 °C using a diffractometer (Miniflex 2000, Rigaku), with CuKα radiation (λ= 1.5418 Å) being obtained by Ni-filtration. Each sample was packed into an Al holder in the range of 5–50 (2Θ).

### Stability studies

The aqueous stability of AZT and their derivatives **2**–**6** was examined at different pH values: pH 1.0 (hydrochloric acid-potassium chloride); pH 2.6 (citric acid-sodium phosphate monobasic); pH 7.4 (sodium phosphate dibasic); pH 9.4 (sodium hydrogen carbonate-sodium hydroxide) and pH 13.2 (potassium chloride-sodium hydroxide). The buffer concentrations were 0.1 mol L^−1^ and the ionic strength (μ) of the buffers was adjusted to 0.1 with calculated amount of sodium chloride (NaCl). All buffer solutions were prepared with Milli Q water. The pH of each solution was determined using a GLP 21 (Crison Instruments) pHmeter. Stock solutions (5 × 10^−5^ mol mL^−1^) of each compound were prepared in DMSO prior to use, with 10 μL of the stock solution being added to 990 μL of buffer, and the vials placed in a water bath at 60 °C during all the experiment. Each vial was withdrawn at predetermined time intervals, and immediately stored at −18 °C until being used. For their analysis, the samples were left to defrost, and the aliquots of 100 μL were sampled and added to 400 μL of MeOH at 25 °C. Then, the solution was analyzed by HPLC to determine the rate of disappearance of each compound.

### HPLC analysis

Samples were analyzed using an HPLC system Agilent^®^ 1100 equipped with Phenomenex^®^ column: Hypersil ODS 5 μ particle diameter (4.6 × 250 mm). Detection was carried out with an UV detector at λ = 267 nm, using the following three solvent systems as mobile phases with a flow rate of 1 mL min^−1^ at 35 °C: A); 50% phosphate buffer (pH 7.5, 0.02 mol L^−1^), 50% MeOH and 2% THF; B) 60% phosphate buffer (pH 7.5, 0.02 mol L^−1^), 40% MeOH and 2% THF; and; C) 65% phosphate buffer (pH 7.5, 0.02 mol L^−1^), 35% MeOH and 2% THF to obtain an adequate peak separation.

## Conclusions

Different techniques were employed to establish preformulation parameters of a series of AZT derivatives. Thus, the acid-basic behavior of ionizable groups was determined to identify the pKa of each analogue as well as most relevant DSC, TG and XRPD characteristic as well as the stability of **2–6** in a wide pH range were also assayed. From an integral analysis of the studies mentioned above and those previously reported (anti HIV activity, cytotoxicity and lipophilicity), we can conclude that compound **3** is an excellent candidate for offering a wide therapeutic potential, and consequently merits further investigation for an integral analysis performing complementary biopharmaceutical studies.

## Figures and Tables

**Tab. 1.** Parameters of the calibration graphs: slope ±SD, intercept ±SD, regression coefficient (*r*), limit of detection (LOD, mmol mL^−1^), quantification (LOQ, mmol mL^−1^) and intra- and inter-day precision (RSD %) for the compounds studied (**1–6**).

**Compound**	**Slope (10^7^) ±SD (10^5^)**	**Intercept ±SD**	***r***	**LOD (10^−7^)**	**LOQ (10^−7^)**	**RSD %**	
						**Intra**	**Inter**
AZT, 1	2.6 ± (8.6)	25.5 ± (28.6)	0.999	3.0	3.1	0.42	0.45
AZT-Tos, 2	3.7 ± (2.0)	1.6 ± (4.8)	0.999	0.6	1.0	0.09	0.38
AZT-Cycl, 3	3.3 ± (6.5)	5.9 ± (31.7)	0.999	2.5	3.0	1.03	1.70
AZT-Py, 4	2.4 ± (5.6)	−2.7 ± (27.3)	0.999	2.1	2.2	0.17	0.36
AZT-Pyp, 5	5.3 ± (2.5)	10.2 ± (16.3)	0.999	8.3	9.8	0.95	1.36
AZT-Ethy, 6	1.1 ± (6.7)	−1.6 ± (44.1)	0.998	7.7	8.9	0.21	0.79

**Tab. 2.** Aqueous stability of AZT (**1**) and their derivatives (**2–6**) for different pHs at 60 °C.

**Compound**	**pH medium**			
	**1.0**	**7.4**	**9.4**	**13.2**
AZT, 1	Stable^a^	Stable^a^	Stable^a^	Stable^a^
AZT-Tos, **2**	Stable^a^	0.94^b^;725.4^c^	14.9^b^; 46.5^c^	15.1^b^; 45.9^c^
AZT-Cycl, **3**	5.43^b^; 128^c^	0.15^b^; 4750^c^	5.58^b^; 124.2^c^	Unstable^d^
AZT-Py, **4**	Stable^a^	Stable^a^	Stable^a^	1.04^b^; 674^c^
AZT-Pyp, **5**	Stable^a^	Stable^a^	Stable^a^	3.23^b^; 238^c^
AZT-Ethy, **6**	Stable^a^	Stable^a^	Stable^a^	1.96^b^; 389^c^

a Stable: degradation products by HPLC were not observed after 80 h of experiment;

b Degradation rate constant, k_obs_ (10^−3^ min^−1^);

c t_½_ min.

d The determination was not possible due to a rapid decomposition.

## References

[1] Broder S, Gallo RC (1984). A pathogenic retrovirus (HTLV-III) linked to AIDS. N Engl J Med.

[2] Mitsuya H, Weinhold KJ, Furman PA, St Clair MH, Lehrman SN, Gallo RC, Bolognesi D, Barry DW, Broder S (1985). 3′-Azido-3′-deoxythymidine (BW A509U): an antiviral agent that inhibits the infectivity and cytopathic effect of human T-lymphotropic virus type III/lymphadenopathy-associated virus in vitro. Proc Natl Acad Sci U S A.

[3] De Clercq E (2009). Anti-HIV drugs: 25 compounds approved within 25 years after the discovery of HIV. Int J Antimicrob Agents.

[4] De Clercq E (2009). The history of antiretrovirals: key discoveries over the past 25 years. Rev Med Virol.

[5] Rautio J, Kumpulainen H, Heimbach T, Oliyai R, Oh D, Jarvinen T, Savolainen J (2008). Prodrugs: design and clinical applications. Nat Rev Drug Discov.

[6] Raviolo MA, Trinchero Hernández JS, Turk G, Briñón MC (2009). Synthesis and antiretroviral evaluation of derivatives of zidovudine. J Braz Chem Soc.

[7] Raviolo MA, Briñón MC (2005). Comparative study of hydrophobicity parameters of novel 5′-carbamates of zidovudine. J Liq Chromatogr Relat Technol.

[8] Moroni GN, Quevedo MA, Ravetti S, Briñón MC (2002). Lipophilic character of novel amino acid derivatives of zidovudine with anti HIV activity. J Liq Chromatogr Relat Technol.

[9] Moroni GN, Bogdanov PM, Briñón MC (2002). Synthesis and in vitro Antibacterial Activity of Novel 5′-O-Analog Derivatives of Zidovudine as Potential Prodrugs. Nucleos Nucleot Nucleic Acids.

[10] Turk G, Moroni GN, Pampuro S, Briñón MC, Salomón H (2002). Antiretroviral Activity and Cytotoxicity of Novel Zidovudine (AZT) Derivatives and the Relation with their Chemical Structure. Int J Antimicrob Agents.

[11] Motura MI, Moroni GN, Teijeiro AS, Salomón H, Briñón MC (2002). 3′-Azido-3′-deoxy-5′-O-isonicotinoylthymidine, a New Prodrug of Zidovudine. Synthesis, Solid State Characterization and Anti HIV-1 Activity. Nucleos Nucleot Nucleic Acids.

[12] Tietgen H, Vogel HG (2006). Physicochemical properties. Drug discovery and evaluation. Safety and pharmacokinetic assay.

[13] Chen XQ, Antman MD, Gesenberg C, Gudmundsson OS (2006). Discovery Pharmaceutics—Challenges and Opportunities. AAPS J.

[14] Steele G, Austin T, Gibson M (2009). Preformulation investigations using small amounts of compound as an aid to candidate drug selection and early development. Pharmaceutical preformulation and formulation.

[15] Avdeef A, Testa B (2002). Physicochemical profiling in drug research: a brief survey of the state-of-the-art of experimental techniques. Cell Mol Life Sci.

[16] Campitelli PA, Velasco MI, Ceppi SB (2006). Chemical and physicochemical characteristics of humic acids extracted from compost, soil and amended soil. Talanta.

[17] Ceppi SB, Velasco MI, De Pauli CP (1999). Differential scanning potentiometry: surface charge development and apparent dissociation constants of natural humic acids. Talanta.

[18] Di L, Kerns EH (2009). Stability challenges in drug discovery. Chem Biodiversity.

[19] Raviolo MA, Sanchez JM, Briñón MC, Perillo MA (2008). Determination of Liposome Permeability of Ionizable Carbamates of Zidovudine by Steady State Fluorescence Spectroscopy. Colloids Surf B.

[20] Christensen JJ, Rytting JH, Izatt RM (1970). Thermodynamics of proton dissociation in dilute aqueous solution. Part XIV. pK, ΔHº, and ΔSº values for proton dissociation from several pyrimidines and their nucleosides at 10 and 40 °C. J Chem Soc B.

[21] Perrin DD, Yalkowsky SH, Sinkula AA, Valvani SC (1980). Physical chemical properties of drugs.

[22] Teijeiro SA, Raviolo MA, Motura MI, Briñón MC (2003). 3′-Azido-3′-deoxy-5′-O-isonicotinoylthymidine, a Novel Antiretroviral Analog of Zidovudine. II Stability in Aqueous Media and Experimental and Theoretical Ionization Constants. Nucleos Nucleot Nucleic Acids.

[23] Norberto FP, Santos SP, Iley J, Silva DB, Corte Real M (2007). Kinetics and mechanism of hydrolysis of benzimidazolylcarbamates. J Braz Chem Soc.

[24] Parang K, Wiebe LI, Knaus EE (1998). Synthesis, in vitro anti-human immunodeficiency virus structure-activity relationships and biological stability of 5′-O-myristoil analogue derivatives of 3′-azido-3′-deoxy-thymidine (AZT) as potential prodrugs. Antiviral Chem Chemother.

